# Sex differences in brain atrophy and cognitive impairment in Parkinson’s disease patients with and without probable rapid eye movement sleep behavior disorder

**DOI:** 10.1007/s00415-021-10728-x

**Published:** 2021-08-03

**Authors:** Javier Oltra, Barbara Segura, Carme Uribe, Gemma C. Monté-Rubio, Anna Campabadal, Anna Inguanzo, Jèssica Pardo, Maria J. Marti, Yaroslau Compta, Francesc Valldeoriola, Alex Iranzo, Carme Junque

**Affiliations:** 1 Medical Psychology Unit, Department of Medicine, Institute of Neurosciences, University of Barcelona, Barcelona, Spain; 2 Institute of Biomedical Research August Pi i Sunyer (IDIBAPS), Barcelona, Catalonia Spain; 3 Centro de Investigación Biomédica en Red Enfermedades Neurodegenerativas (CIBERNED: CB06/05/0018-ISCIII), Barcelona, Catalonia Spain; 4 Research Imaging Centre, Campbell Family Mental Health Research Institute, Centre for Addiction and Mental Health (CAMH), University of Toronto, Toronto, Canada; 5 Parkinson’s Disease and Movement Disorders Unit, Neurology Service, Hospital Clínic de Barcelona, Institute of Neurosciences, University of Barcelona, Barcelona, Catalonia Spain; 6 Sleep Disorders Center, Neurology Service, Hospital Clínic, Barcelona, Catalonia Spain

**Keywords:** Parkinson’s disease, Sex differences, REM sleep behavior disorder, Magnetic resonance imaging, Gray matter atrophy, Cognitive impairment

## Abstract

**Background:**

The presence of rapid eye movement sleep behavior disorder (RBD) contributes to increase cognitive impairment and brain atrophy in Parkinson’s disease (PD), but the impact of sex is unclear. We aimed to investigate sex differences in cognition and brain atrophy in PD patients with and without probable RBD (pRBD).

**Methods:**

Magnetic resonance imaging and cognition data were obtained for 274 participants from the Parkinson's Progression Marker Initiative database: 79 PD with pRBD (PD-pRBD; male/female, 54/25), 126 PD without pRBD (PD-non pRBD; male/female, 73/53), and 69 healthy controls (male/female, 40/29). FreeSurfer was used to obtain volumetric and cortical thickness data.

**Results:**

Males showed greater global cortical and subcortical gray matter atrophy than females in the PD-pRBD group. Significant group-by-sex interactions were found in the pallidum. Structures showing a within-group sex effect in the deep gray matter differed, with significant volume reductions for males in one structure in in PD-non pRBD (brainstem), and three in PD-pRBD (caudate, pallidum and brainstem). Significant group-by-sex interactions were found in Montreal Cognitive Assessment (MoCA) and Symbol Digits Modalities Test (SDMT). Males performed worse than females in MoCA, phonemic fluency and SDMT in the PD-pRBD group.

**Conclusion:**

Male sex is related to increased cognitive impairment and subcortical atrophy in de novo PD-pRBD. Accordingly, we suggest that sex differences are relevant and should be considered in future clinical and translational research.

**Supplementary Information:**

The online version contains supplementary material available at 10.1007/s00415-021-10728-x.

## Introduction

There is significant cumulative evidence for Alzheimer’s disease [1] and Parkinson’s disease (PD) [2, 3] that susceptibility to regional brain atrophy and cognitive impairment differs by sex. These between-sex differences on brain degeneration have implications for implementing prevention, diagnosis, and treatment strategies in the context of precision medicine.

Early population-based studies report that males have a two-fold increased risk of developing PD [4]. Males with PD, in comparison to females, also have decreased performance in global cognition [5–8], memory [6–8], verbal fluency [5, 7–9], processing speed [7, 9], and inhibition [9]. In contrast, females with PD have greater impairment in visuospatial function than males [6–8]. A recent meta-analysis revealed greater frontal executive deficit in males than females [10]. In addition, male sex is associated with cognitive impairment [11] and with progression to mild cognitive impairment (MCI) [8, 12] as well as dementia [12]. Male sex is an established predictor of progressive cognitive decline [13].

Structural magnetic resonance imaging (MRI) studies have also evidenced sex-based differences in PD. Males have pronounced cortical thinning in frontal, parietal, temporal, and occipital regions compared with females [14]. Greater tissue loss in males with de novo PD has also been described in some cortical regions and in the left thalamus by deformation-based morphometry [15]. Studies have also identified disrupted structural connectivity in PD males compared to PD females [14, 15].

Isolated rapid eye movement behavior disorder (RBD) is a well-known prodrome of the synucleinopathies, with a rate of conversion of 90% after 15-year follow-up [16]. For unknown reasons, about 80% of the patients diagnosed in sleep centers with isolated RBD are of male sex [17]. PD patients have a prevalence of RBD symptomatology of around 40% [18]. The presence of probable RBD (pRBD) in PD has also been associated with more severe cognitive impairment in patients with de novo PD [19, 20], with a greater degree of cognitive decline over time [19]. Moreover a higher prevalence of MCI has been reported in PD patients with polysomnographic diagnosis of RBD [21]. Structural MRI studies in patients with de novo PD indicate that cortical [22] and subcortical volumes [22, 23] are reduced in groups with pRBD compared to groups without pRBD. To the best of our knowledge, no previous studies have investigated the impact of sex differences on brain atrophy and cognitive deficits in patients with PD and pRBD.

In the current work, we aimed to explore sex differences in brain and cognition in a large sample of newly diagnosed drug-naïve patients with PD, with and without probable RBD (PD-pRBD and PD-non pRBD groups, respectively). We hypothesized that the between-sex differences would be more marked in the PD-pRBD group than in the PD-non pRBD due to a greater degree of neurodegeneration.

## Methods

### Participants

Data were obtained from the Parkinson’s Progression Markers Initiative database (PPMI, http://www.ppmi-info.org) [24], including T1-weighted images, clinical information, and neuropsychological data from 205 patients with PD and 69 healthy controls. The PD cohort was then divided into four groups by their sex and pRBD status, the latter of which was established based on a five-point cutoff on the RBD Screening Questionnaire (RBDSQ) [25]. The final sample comprised 6 groups: 73 PD-non pRBD males, 53 PD-non pRBD females, 54 PD-pRBD males, 25 PD-pRBD females, 40 control males, and 29 control females.

The inclusion criteria were as follows: (i) recent diagnosis of PD with asymmetric resting tremor or asymmetric bradykinesia, or two from among bradykinesia, resting tremor, and rigidity; (ii) absence of PD treatment; (iii) neuroimaging evidence of significant dopamine transporter deficit consistent with a clinical diagnosis of PD, and excluding conditions that can mimic PD, such as drug-induced and vascular parkinsonism or essential tremor; (iv) T1-weighted images available (PD and control groups); and (v) age older than 50 and younger than 85 years old (PD and control groups). The exclusion criteria for all participants were as follows: (i) diagnosis of dementia; (ii) significant psychiatric, neurologic, or systemic comorbidity; (iii) a first-degree family member with PD; and (iv) presence of MRI motion artifacts, field distortions, intensity inhomogeneities, or detectable structural brain lesions. The sample selection process is shown in Supplementary Fig. 1.

### Clinical and neuropsychological assessments

A detailed clinical assessment was performed. This included measurements of PD symptoms by the Movement Disorders Society Unified PD Rating Scale (MDS-UPDRS), PD motor symptoms by the MDS-UPDRS motor section (Part III), disease severity by the Hoehn and Yahr scale (H&Y), global cognition by the Montreal Cognitive Assessment (MoCA), depressive symptoms by the 15-item Geriatric Depression Scale (GDS-15), olfactory function by the University of Pennsylvania Smell Identification Test (UPSIT-40), probable RBD status and symptomatology by the RBDSQ, and excessive daytime sleepiness by the Epworth Sleepiness Scale (ESS) [24]. All subjects also underwent a neuropsychological battery that included the following: phonemic (letter ‘f’) and semantic (animals, fruits and vegetables) verbal fluency tests; the Symbol Digit Modalities Test (SDMT); Letter-Number Sequencing (LNS); the Benton Judgment of Line Orientation short form (JLO), 15-item version; and the Hopkins Verbal Learning Test-Revised (HVLT-R) [24]. All neuropsychological data were reported using z scores calculated based on the control group's means and standard deviations.

### MRI images

T1-weighted MRI scans were acquired using 1.5 or 3-Tesla scanners at different centers using magnetization prepared rapid gradient echo imaging (MPRAGE) sequences. Typical MRI parameters were as follows: repetition time = 5–11 ms; echo time = 2–6 ms; slice thickness 1–1.5 mm; inter-slice gap 0 mm; voxel size 1 × 1 × 1.2 mm; matrix 256 × 160 minimum. Details can be found at http://www.ppmi-info.org/wp-content/uploads/2010/07/Imaging-Manual.pdf. There were no differences in the distribution of 1.5 and 3-Tesla images across groups (Supplementary Table 1).

Cortical thickness was estimated using the automated processing stream and specific segmentation tools of FreeSurfer (version 6.0, http://surfer.nmr.mgh.harvard.edu). Uribe et al. provide a detailed description about processing with the FreeSurfer stream [26]. After preprocessing, results for each subject were inspected visually to ensure the accuracy of registration, skull stripping, segmentation, and cortical surface reconstruction. Errors were fixed by manual intervention following standard procedures (see applied manual interventions in Supplementary Fig. 1). Deep gray matter (GM) mean volumes (i.e., in the thalamus, putamen, pallidum, caudate, hippocampus, amygdala, accumbens, and brainstem) and total cortical and subcortical GM were extracted [27]. First, volumes were made bilateral by averaging those of the left and right hemisphere as [(left volume + right volume)/2]. Second, volume ratios were calculated using the estimated total intracranial volume (eTIV) to perform global and partial volumetric analyses [(volume/eTIV) × 100]. Thus, significant eTIV between-sex differences in the three groups were already controlled in the subsequent analyses (Supplementary Table 2).

### Statistical analyses

The main effects of group and sex were computed for the demographic variables by two-way analysis of variance (ANOVA) followed by Bonferroni post hoc tests to analyze sex differences in group conditions. These analyses revealed that males were significantly older than females in the control group; consequently, subsequent analyses that involved this group included age as a covariate (Table 1). No significant main effect of group was found by age (*F* = 1.892, *p* = 0.153), education (*F* = 2.959, *p* = 0.054), age of disease onset (*F* = 3.264, *p* = 0.072), or PD duration (*F* = 0.045, *p* = 0.832). There were no differences in the sex distribution across the PD groups and healthy controls (Chi-squared = 2.558, *p* = 0.278).

**Table 1:** Demographic and clinical characteristics of HC, PD-non pRBD, and PD-pRBD females and males

	HC	PD-non pRBD	PD-pRBD	Sex main effect test stat (*P* value)
Age, years				
F	60.6 (5.9)	60.9 (7.4)	63.5 (7.5)	6.215 (0.013)*
M	64.1 (7.1)	63.2 (7.4)	64.7 (7.0)	
Education, years				
F	16.2 (2.9)	15.4 (2.9)	15.2 (3.1)	3.141 (0.077)
M	17.0 (2.5)	15.8 (3.0)	16.1 (2.9)	
Age of onset, years				
F	NA	60.0 (7.4)	62.6 (7.6)	2.348 (0.127)
M	NA	62.3 (7.2)	63.6 (6.9)	
PD duration, months				
F	NA	10.8 (8.5)	9.9 (6.8)	0.278 (0.599)
M	NA	10.3 (6.2)	11.6 (7.2)	
MDS-UPDRS				
F	NA	28.4 (10.0)	32.0 (11.9)	3.774 (0.053)
M	NA	30.1 (11.3)	37.4 (14.1)	
MDS-UPDRS Part III				
F	NA	19.0 (7.6)	17.9 (7.6)	7.371 (0.007)**
M	NA	20.4 (8.3)	23.3 (9.4)	
H&Y stage, *n*, 1/2/3				
F	NA	1.7 (2.1)	2.6 (2.5)	0.002 (0.967)
M	NA	35/37/1	21/33/0	
GDS-15				
F	1.9 (3.2)	1.7 (2.1)	2.6 (2.5)	0.002 (0.967)
M	1.2 (2.8)	2.3 (2.2)	2.7 (2.4)	
ESS				
F	4.6 (2.9)	4.9 (3.4)	6.8 (3.6)	0.822 (0.365)
M	5.3 (3.5)	5.7 (2.9)	6.4 (3.7)	
RBDSQ				
F	1.5 (1.2)	2.7 (1.1)	6.0 (1.4)	7.405 (0.007)**
M	1.9 (1.4)	2.6 (1.1)	7.2 (1.9)	
UPSIT				
F	34.9 (3.4)	23.8 (8.6)	21.4 (8.7)	3.401 (0.066)
M	33.6 (4.1)	21.0 (7.4)	18.7 (8.0)	

Data are presented by groups as mean (SD), except for H&Y. Two-way analyses of variance (ANOVA) followed by Bonferroni post hoc tests were used for all demographic variables. Two-way analyses of covariance (ANCOVA) with age as covariable, followed by Bonferroni post hoc tests were used for all clinical variables. Except for MDS-UPDRS, that was analyzed by two-way analysis of variance (ANOVA); and H&Y, by Pearson’s Chi-squared

*ESS* Epworth Sleepiness Scale; *F* female; *GDS*-*15* the 15-item Geriatric Depression Scale; HC healthy controls; *H&Y* Hoehn and Yahr scale; *M* male; *MDS*-*UPDRS* Movement Disorder Society Unified Parkinson’s Disease Rating Scale; *PD-non pRBD* PD without probable RBD; *PD-pRBD* PD with probable RBD; *RBDSQ* REM Sleep Behavior Disorder Screening Questionnaire; *UPSIT* University of Pennsylvania Smell Identification Test

*Sex differences in HC group (*P* < 0.05)

**Sex differences in PD-pRBD group (*P* < 0.05)

The group-by-sex interaction for clinical, neuropsychological, volumetric, and mean cortical thickness variables was assessed by two-way ANOVA or covariance (ANCOVA), followed by Bonferroni post hoc tests, as appropriate. Pearson’s Chi-squared test was used to analyze differences in categorical measures.

Additionally, we explored the within-group sex effect of neuropsychological, mean cortical thickness and volumetric variables. First, we regressed out the effect of normal aging and sex. Expected z scores adjusted for age and sex were calculated based on a multiple regression analysis performed in the HC group and subtracted from the observed variables [28]. Second, within-group sex effects and group-by-sex interactions were explored by two-way ANOVA followed by Bonferroni post hoc tests. Lastly, the between-group differences regarding the within-group sex effects were estimated to explore the significant group-by-sex interactions. The statistical significance threshold was set at* p* < 0.05 and all analyses were performed with IBM SPSS Statistics 27.0.0 (2020; IBM Corp., Armonk, NY).

Intergroup comparisons of cortical thickness were performed using a vertex-by-vertex general linear model in FreeSurfer v6.0. The model included cortical thickness as a dependent factor, group as an independent factor, and demeaned age as covariable. All results were corrected for multiple comparisons using a pre-cached cluster-wise Monte Carlo simulation with 10,000 iterations. Reported cortical regions reached a two-tailed corrected significance level of *p* < 0.05.

## Results

### Clinical characteristics

A significant sex effect was found with motor severity (MDS-UPDRS Part III) and RBD (RBDSQ). There was a significant group-by-sex interaction in the RBDSQ score (*F* = 4.749, *p* = 0.009), with post hoc analyses also showing that males in the PD-pRBD group had more severe motor and RBD symptoms than females in this group. No significant main effect of sex was found in the global MDS-UPDRS score, the H&Y stage, GDS-15, ESS, and UPSIT scores (Table 1).

### Neuropsychological performance

**Fig. 1:**
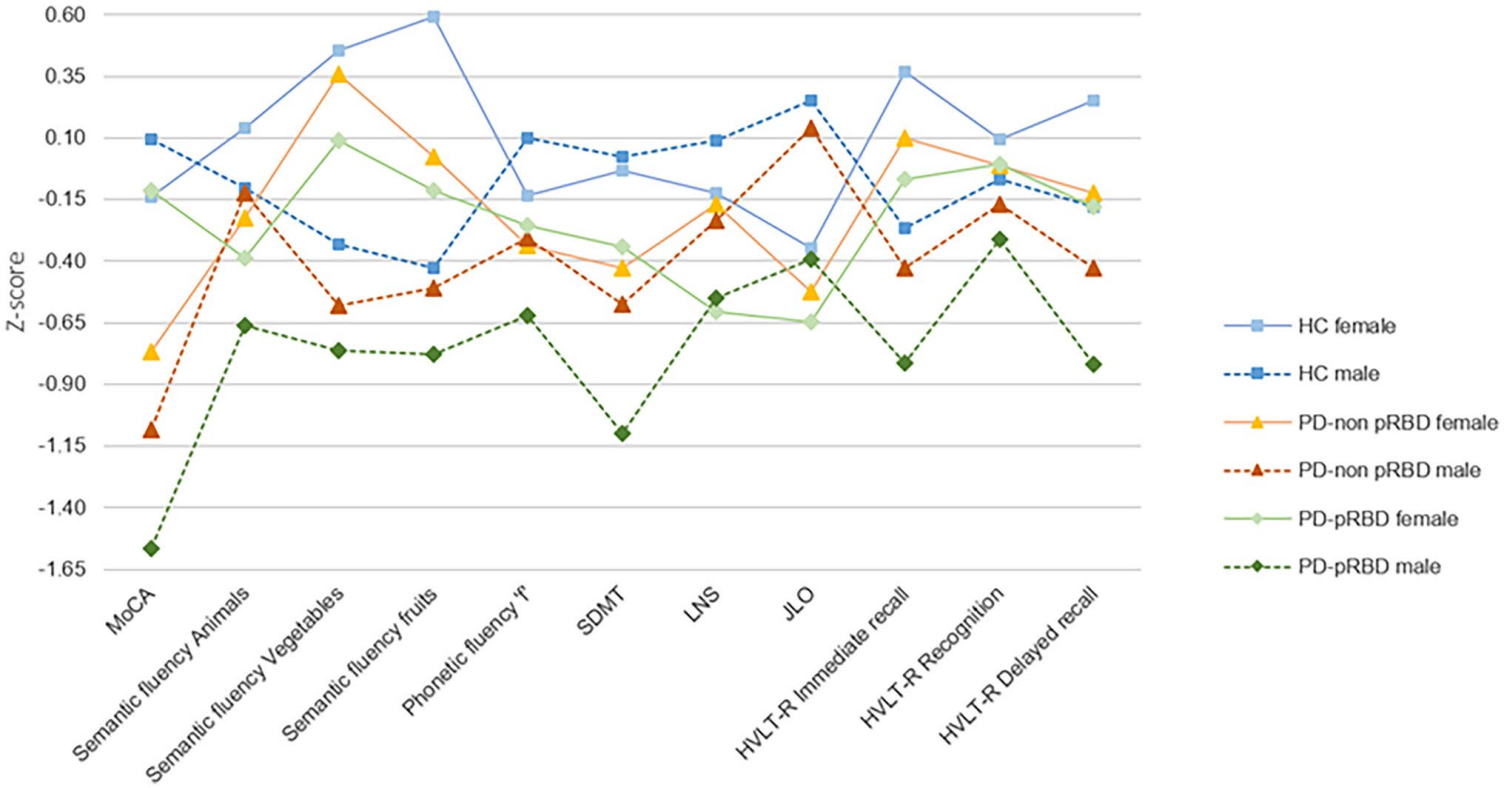
Neuropsychological performance. Tasks are indicated in the *x* axis. Group means in each task are presented as *z* scores, as indicate in *y* axis. Lower *z* scores indicate worse performance. Descriptive statistics, as mean (SD), are available in Supplementary Table 3. Healthy controls in blue, PD-non pRBD in warm colors, PD-pRBD in green; lighter for females and darker for males. HC represented by filled squares, PD-non pRBD by filled triangles and PD-pRBD by filled rhombuses. Females by a continuous line and males by a dashed line. Data are presented as *z* scores. Abbreviations:  *MoCA* Montreal Cognitive Assessment; *SDMT* Symbol Digit Modalities Test; *LNS* Letter-Number Sequencing; *JLO* Benton Judgment of Line Orientation; *HVLT*-*R* Hopkins Verbal Learning Test-Revised, *HC* healthy controls; *PD-non pRBD* PD without probable RBD; *PD-pRBD* PD with probable RBD

A significant group-by-sex interaction was found in the MoCA (*F* = 4.758, *p* = 0.009) and SDMT (*F* = 4.196, *p* = 0.016). Both groups of PD males performed worse than HC males in MoCA and SDMT, PD-pRBD males performed worse than PD-non pRBD males in SDMT (Fig. 1 and Supplementary Table 3).

Complementary, significant within-group sex effects were found in the MoCA, phonemic fluency and SDMT in the PD-pRBD group after regressing out age and sex, in which males performed lower than females (Supplementary Table 4). A significant within-group sex effect in semantic fluency (fruits) was observed in the PD-non pRBD group, with lower performance in females than males. No within-group sex effect was observed in the control group. Significant group-by-sex interactions remained after controlling the effect of normal aging and sex (Supplementary Tables 4 and 5). Between-groups differences regarding the within-group sex effects in MoCA and SDMT showed significant differences between PD-pRBD and the other two groups (Supplementary Table 5).

### MRI volumetry

We did not find any vertex-wise sex effects in cortical thickness. Regarding subcortical volumetry, there was a significant group-by-sex interaction in the bilateral pallidum (*F* = 3.084, *p* = 0.047). Post hoc analyses showed that PD-pRBD males had smaller pallidum volume than PD-non pRBD males (Table 2).

**Table 2:** Magnetic resonance imaging derived measures of HC, PD-non pRBD, and PD-pRBD females and males

	HC	PD-non pRBD	PD-pRBD	Group-by-sex test stat (*P* value)
Global atrophy				
Cortical GM				
F	30.11 (1.80)	29.39 (2.23)	30.03 (2.34)	1.577 (0.209)
M	29.39 (2.26)	28.15 (1.98)	28.15 (2.39)	
Subcortical GM				
F	3.69 (0.27)	3.63 (0.26)	3.66 (0.32)	1.773 (0.172)
M	3.54 (0.28)	3.51 (0.24)	3.41 (0.24)	
Mean CTh, mm				
F	2.44 (0.10)	2.42 (0.10)	2.41 (0.09)	0.017 (0.984)
M	2.41 (0.12)	2.39 (0.12)	2.38 (0.12)	
Deep GM nuclei				
Thalamus				
F	0.460 (0.030)	0.465 (0.042)	0.456 (0.044)	0.741 (0.478)
M	0.443 (0.046)	0.441 (0.040)	0.429 (0.037)	
Caudate				
F	0.222 (0.028)	0.221 (0.027)	0.229 (0.022)	2.047 (0.131)
M	0.215 (0.024)	0.212 (0.025)	0.207 (0.024)	
Putamen				
F	0.303 (0.038)	0.294 (0.035)	0.296 (0.039)	0.995 (0.371)
M	0.290 (0.034)	0.286 (0.029)	0.275 (0.035)	
Pallidum				
F	0.126 (0.013)	0.127 (0.014)	0.131 (0.015)	3.084 (0.047)*
M	0.124 (0.015)	0.127 (0.013)	0.120 (0.015)	
Hippocampus				
F	0.279 (0.028)	0.267 (0.030)	0.272 (0.031)	0.802 (0.449)
M	0.257 (0.026)	0.252 (0.028)	0.247 (0.025)	
Amygdala				
F	0.110 (0.013)	0.103 (0.015)	0.108 (0.022)	2.397 (0.093)
M	0.110 (0.014)	0.105 (0.013)	0.101 (0.013)	
Accumbens				
F	0.032 (0.006)	0.031 (0.007)	0.034 (0.007)	1.618 (0.200)
M	0.031 (0.004)	0.030 (0.006)	0.030 (0.006)	
Brainstem				
F	1.382 (0.100)	1.412 (0.128)	1.412 (0.110)	2.393 (0.093)
M	1.390 (0.134)	1.375 (0.113)	1.338 (0.128)	

Data are presented by groups as mean (SD). Volumetric variables are presented in ratios estimated by ((volume/eTIV) × 100). Two-way analyses of covariance (ANCOVA) with age as covariable, followed by Bonferroni post hoc tests, were used for all variables

*CTh *cortical thickness; *F* female; *GM* gray matter; *HC* healthy controls; *M* male; *PD-non pRBD* PD without probable RBD; *PD-pRBD* PD with probable RBD

*Differences between PD-non pRBD males and PD-pRBD males (*P* < 0.05)

Supplementary analysis, after regressing out age and sex, showed that in the PD-pRBD group males had smaller global cortical and subcortical GM volumes than females. Furthermore, males had significantly smaller volume than females in three subcortical structures in the PD-pRBD group (caudate, pallidum and brainstem) versus in one in the PD-non pRBD group (brainstem), and in none in the control group (Supplementary Table 6). Significant group-by-sex interaction remained after controlling the effect of normal aging and sex (Supplementary Tables 5 and 6). Between-groups differences regarding the within-group sex effects showed a significant difference between PD groups and a trend to significance between PD-pRBD and HC in the pallidum. As expected, there were no differences between PD-non pRBD and HC (Supplementary Table 5).

Significant group-by-sex interactions in neuropsychological (MoCA) and MRI (pallidum) measures remain significant after controlling by motor disease severity (Supplementary Tables 7 and 8).

In summary, we showed a significant interaction in pallidum, showing smaller pallidum volume in PD-pRBD males than in PD-non pRBD males. Additionally, PD-pRBD males showed smaller global cortical and subcortical GM volumes than females, as well as, a different number of structures showing within-group sex differences. This applied to no structure in the control group, one in the PD-non-pRBD group, and three in the PD-pRBD group. In all cases, males showed decreased volumes compared with females.

## Discussion

Among drug-naïve patients, in the PD-pRBD group males had more severe motor and RBD symptomatology, worse cognitive performance, and greater subcortical volume atrophy than females. Such sex differences were also observed in subcortical volumes in PD-non pRBD group, but to a greater extent in the former.

Clinically, despite a similar age at the time of the study, age of disease onset and PD duration, males in the PD-pRBD group had greater motor impairment and more RBD symptoms. Cognitive impairment was also greater in males in the PD-pRBD group. We found a significant group-by-sex interaction in MoCA and SDMT. Notably, the sex effects in MoCA and SDMT were greater in the PD-pRBD group compared with the other two groups. Specifically, we found that males performed significantly worse than females in MoCA, phonemic fluency and SDMT in the PD-pRBD group. By contrast, females in the PD-non pRBD showed greater impairment only in one semantic fluency test than males. These results suggest that, if PD patients with RBD symptomatology, showed sex differences consistent with those of previous studies in drug-naïve patients with PD as a whole group, showing that males have greater global cognitive impairment [6, 7], verbal fluency, and processing speed [7] impairments. In contrast, those differences mostly disappear, in the PD-non pRBD group.

Global MRI measures revealed smaller total cortical and subcortical GM volumes in males of the PD-pRBD group, but not in the PD-non pRBD and control groups. This finding suggesting that, just like the atrophy patterns are more severe in PD with RBD symptomatology [22, 23], the sex differences are more marked in this PD subtype.

It has been reported that males, even adjusting for brain size, have larger volumes in several structures than females [29, 30]. Nonetheless, more accelerated aging effects have been described in males in regional volumes [31, 32]. In summary, aging seems to reverse sex-related structural differences in the brain, probably due to hormonal effects, resulting in a greater vulnerability of males to brain atrophy, especially in degenerative conditions.

In addition to the greater global atrophy among males compared with females, we also found vulnerability to differential volumetric atrophy by sex among various subcortical structures. There was increased subcortical atrophy in males compared with females in both PD groups, and we observed that sex differences in subcortical regions were more evident in the PD-pRBD group, with a significant group-by-sex interaction in the pallidum. The sex effect in the pallidum was greater in the PD-pRBD group compared with the PD-non pRBD group. Specifically, we found significant differences in three structures in the PD-pRBD group compared with only one structures in the PD-non pRBD group and none structure in the control group. In de novo PD as a whole group, using voxel-based morphometry, males have been shown to have increased atrophy in the left thalamus compared to females [15]. However, following the applied classification, the PD-pRBD group showed sex differences in the bilateral pallidum, caudate, and brainstem; but the PD-non pRBD group showed only one sex difference, in the brainstem.

Together, our results provide evidence for the presence of sex differences in cognition and brain structure following a continuum from normal aging to patients with PD and pRBD. In both PD groups, males show more severe atrophy, suggesting that female sex confers protective benefits against neurodegeneration. Several pathophysiological mechanisms have been suggested as being responsible for sex differences in neurodegenerative processes. Dysregulated gene expression and sex hormones have been related to these sex differences in the pathophysiology of PD, including vulnerability of the dopaminergic system, neuroinflammation, and oxidative stress [2]. Another implicated mechanism is the alpha-synuclein, that has been observed in more decreased plasma concentrations in males than females in advanced stages of PD; and its concentration has been associated with cognitive impairment and sleep disorders in PD males [33]. By analyzing the subcortical structural volumes of 38,851 subjects, several genes involved in the regulation of neuronal apoptosis, inflammation/immunity, and susceptibility to neurological disorders have been identified [34]. Nevertheless, in the future, other functional biomarkers and imaging techniques are needed to investigate the specific mechanisms underlying sex-related brain differences in PD.

The main strength of the paper is a very large sample that allows testing sex effects on brain and cognition in PD and the main limitation of our study is using a validated RBD questionnaire instead of a confirmed polysomnography diagnosis. In this sense, the RBDSQ showed a sensitivity of 0.47 and a specificity of 0.78 in a cohort of PD de novo patients [35]. The use of a questionnaire could increment the false positive discovery rate by overestimating the incidence of clinically significant RBD symptomatology and limit the generalisability of the obtained results. Another limitation is that PPMI data were acquired from a multicenter cohort having differences in MRI acquisition. Finally, we could not have a group of healthy controls with probable RBD to take into account the influence of this condition isolated.

In summary, our results underpin the role of sex as being important to understanding the phenotypic expression of PD. Our findings also indicate that sex male is related to increased functional alterations in motor, RBD, and cognitive domains among drug-naïve PD patients with pRBD. Also, PD-pRBD male patients show more atrophy in subcortical structures than PD-pRBD females and these sex differences are in more structures than in patients without pRBD. Accordingly, we suggest that sex differences are relevant factors to be considered in clinical trials.

## Supplementary Information

Below is the link to the electronic supplementary material.

Supplementary file1 (DOCX 137 KB)

## Data Availability

Data used in the preparation of this article were obtained from the Parkinson’s Progression Markers Initiative (PPMI) database (www.ppmi-info.org/data). For up-to-date information on the study, visit www.ppmi-info.org.
