# Supplementary material for: Sex differences in brain atrophy and cognitive impairment in Parkinson’s disease patients with and without probable rapid eye movement sleep behavior disorder
Source: J Neurol. 2021 Aug 3;269(3):1591–9. doi: 10.1007/s00415-021-10728-x (PMC8857118; doi:10.1007/s00415-021-10728-x)
Supplement: Supplementary file 1 — Supplementary file1 (DOCX 137 KB) [file 415_2021_10728_MOESM1_ESM.docx]

**Sex differences in brain atrophy and cognitive impairment in Parkinson’s disease patients with and without probable rapid eye movement sleep behavior disorder**

**Journal of Neurology**

Javier Oltra^a, b^, Barbara Segura^a, b, c*^, Carme Uribe^a, b, d^, Gemma C. Monté-Rubio^a^, Anna Campabadal^a, b^, Anna Inguanzo^a, b^, Jèssica Pardo^a^, Maria J. Martí^b,c,e,^ , Yaroslau Compta^b, c, e^, Francesc Valldeoriola^b, c, e^, Alex Iranzo^b, c, f^, Carme Junque^a, b, c^

^a^Medical Psychology Unit, Department of Medicine, Institute of Neurosciences, University of Barcelona, Barcelona, Catalonia, Spain

^b^ Institute of Biomedical Research August Pi i Sunyer (IDIBAPS), Barcelona, Catalonia, Spain

^c^Centro de Investigación Biomédica en Red Enfermedades Neurodegenerativas (CIBERNED: CB06/05/0018-ISCIII), Barcelona, Catalonia, Spain

^d^Research Imaging Centre, Campbell Family Mental Health Research Institute, Centre for Addiction and Mental Health (CAMH), University of Toronto, Toronto, Canada

^e^ Parkinson's Disease & Movement Disorders Unit, Neurology Service, Hospital Clínic de Barcelona, Institute of Neurosciences, University of Barcelona, Barcelona, Catalonia, Spain

^f^Sleep Disorders Center, Neurology Service, Hospital Clínic, Barcelona, Catalonia, Spain

^*^Corresponding author. Dr Barbara Segura. Medical Psychology Unit, Department of Medicine, University of Barcelona, Casanova 143, 08036, Barcelona, Spain.  phone [+34] 934039297 // 93 4034446 Fax: [+34] 93 4035294. E-mail addres: bsegura@ub.edu (B.Segura).

**Supplementary Table 1**

MRI field strength distribution of the groups

|  | **1.5 T** | **3 T** | **Test stat**  **(P value)** |
| --- | --- | --- | --- |
| HC female | 8 (27.6%) | 21 (72.4%) | 3.913 (0.562) |
| HC male | 6 (15.0%) | 34 (85.0%) |  |
| PD-non pRBD female | 15 (28.3%) | 38 (71.7%) |  |
| PD-non pRBD male | 22 (30.1%) | 51 (69.9%) |  |
| PD-pRBD female | 5 (20.0%) | 20 (80.0%) |  |
| PD-pRBD male | 15 (27.8%) | 39 (72.2%) |  |

Data are presented by groups as n (%). Pearson's chi-squared was used.

Abbreviations: HC = healthy controls; PD-non pRBD = PD without probable RBD; PD-pRBD = PD with probable RBD; T = Tesla.

**Supplementary Table 2**

Estimated total intracranial volume of HC, PD-non pRBD, and PD-pRBD females and males

|  |  | **HC** | **PD-non pRBD** | **PD-pRBD** | **Sex main effect**  **test stat (P value)** |
| --- | --- | --- | --- | --- | --- |
| eTIV, cm^3^ | F | 1418.4  (112.5) | 1480.9  (138.1) | 1442.2  (132.9) | 111.003 (<.0.001) ^a, b, c^ |
|  | M | 1589.5  (154.8) | 1682.2  (134.0) | 1651.6  (164.2) |  |

Data are presented by groups as mean (SD). Two-way analyses of variance (ANOVA), followed by Bonferroni post-hoc tests, were used.

^a^ Sex differences in HC group; ^b^ Sex differences in PD-non pRBD group; ^c^ Sex differences in PD-pRBD group (P < 0.05)

Abbreviations: F= female; eTIV = estimated total intracranial volume; HC = healthy controls; M = male; PD-non pRBD = PD without probable RBD; PD-pRBD = PD with probable RBD.

|  | |  | **HC** | **PD-non pRBD** | **PD-pRBD** | **Group-by-sex**  **test stat (P value)** |
| --- | --- | --- | --- | --- | --- | --- |
| MoCA | | F | -0.14 (0.90) | -0.77 (1.81) | -0.11 (1.52) | 4.758 (0.009) ^a, b^ |
|  | | M | 0.10 (1.07) | -1.08 (1.93) | -1.56 (1.81) |  |
| Semantic fluency | |  |  |  |  |  |
|  | Animals | F | 0.14 (0.97) | -0.23 (0.94) | -0.39 (0.75) | 1.270 (0.283) |
|  |  | M | -0.10 (1.02) | -0.12 (0.97) | -0.66 (0.84) |  |
|  | Vegetables | F | 0.46 (1.03) | 0.36 (1.19) | 0.09 (0.88) | 0.156 (0.856) |
|  |  | M | -0.33 (0.84) | -0.58 (1.05) | -0.76 (1.06) |  |
|  | Fruits | F | 0.59 (0.81) | 0.03 (0.95) | -0.11 (0.80) | 1.379 (0.254) |
|  |  | M | -0.43 (0.91) | -0.51 (0.96) | -0.78 (0.95) |  |
| Phonetic fluency ‘f’ | | F | -0.13 (0.92) | -0.34 (1.04) | -0.25 (0.84) | 1.577 (0.209) |
|  | | M | 0.10 (1.06) | -0.31 (1.03) | -0.62 (0.95) |  |
| SDMT | | F | -0.03 (0.86) | -0.43 (0.98) | -0.34 (0.86) | 4.196 (0.016) ^a, b, c^ |
|  | | M | 0.02 (1.10) | -0.58 (0.89) | -1.10 (1.11) |  |
| LNS | | F | -0.12 (0.91) | -0.17 (0.92) | -0.61 (0.88) | 0.548 (0.579) |
|  | | M | 0.09 (1.06) | -0.23 (1.09) | -0.55 (0.95) |  |
| JLO | | F | -0.35 (1.13) | -0.52 (1.08) | -0.65 (1.39) | 0.917 (0.401) |
|  | | M | 0.25 (0.82) | 0.14 (1.03) | -0.39 (1.27) |  |
| HLVT-R | |  |  |  |  |  |
|  | Immediate recall | F | 0.37 (0.90) | 0.10 (1.03) | -0.07 (0.89) | 0.293 (0.746) |
|  |  | M | -0.26 (1.00) | -0.43 (1.18) | -0.82 (1.07) |  |
|  | Recognition | F | 0.09 (0.88) | -0.01 (0.90) | -0.01 (0.76) | 0.162 (0.851) |
|  |  | M | -0.07 (1.09) | -0.17 (0.91) | -0.31 (0.98) |  |
|  | Delayed recall | F | 0.25 (0.77) | -0.13 (0.94) | -0.18 (0.94) | 0.655 (0.520) |
|  |  | M | -0.18 (1.11) | -0.43 (1.04) | -0.82 (1.09) |  |

**Supplementary Table 3**

Neuropsychological tasks scores of HC, PD-non pRBD, and PD-pRBD females and males

Data are presented in z-scores by groups as mean (SD). Two-way analyses of covariance (ANCOVA) with age as covariable, followed by Bonferroni post-hoc tests, were used for all variables.

^a^ Differences between HC males and PD-non pRBD males, ^b^ Differences between HC males and PD-pRBD males, ^c^ Differences between PD-non pRBD males and PD-pRBD males (P < 0.05).

Abbreviations: F= female; HC = healthy controls; HVLT-R = Hopkins Verbal Learning Test-Revised; JLO = Benton Judgment of Line Orientation; LNS = Letter-Number Sequencing; M = male; MoCA = Montreal Cognitive Assessment; PD-non pRBD = PD without probable RBD; PD-pRBD = PD with probable RBD; SDMT = Symbol Digit Modalities Test.

|  | |  | **HC** | **PD-non pRBD** | **PD-pRBD** | **Group-by-sex**  **test stat (P value)** |
| --- | --- | --- | --- | --- | --- | --- |
| MoCA | | F | -0.01 (0.91) | -0.64 (1.82) | 0.05 (1.53) | 4.828 (0.009)^a, b^ |
|  | | M | -0.01 (1.06) | -1.20 (1.93) | -1.66 (1.79) |  |
| ***Within-group sex effect, test stat (P value)*** | |  | ***0.000 (0.998)*** | ***3.584 (0.059)*** | ***18.419 (<0.001)*** |  |
| Semantic fluency | |  |  |  |  |  |
|  | Animals | F | -0.01 (0.96) | -0.38 (0.97) | -0.57 (0.79) | 0.963 (0.383) |
|  |  | M | -0.01 (1.02) | -0.05 (0.94) | -0.57 (0.85) |  |
|  | ***Within-group sex effect, test stat (P value)*** |  | ***0.000 (.997)*** | ***3.607 (0.059)*** | ***0.000 (0.985)*** |  |
|  | Vegetables | F | 0.02 (1.02) | -0.08 (1.17) | -0.32 (0.87) | 0.137 (0.872) |
|  |  | M | 0.02 (0.85) | -0.24 (1.04) | -0.41 (1.05) |  |
|  | ***Within-group sex effect, test stat (P value)*** |  | ***0.000 (0.996)*** | ***0.751 (0.387)*** | ***0.751 (0.387)*** |  |
|  | Fruits | F | -0.01 (0.80) | -0.57 (0.93) | -0.70 (0.78) | 1.514 (0.222) |
|  |  | M | -0.01 (0.86) | -0.09 (0.96) | -0.36 (.94) |  |
|  | ***Within-group sex effect, test stat (P value)*** |  | ***0.000 (0.999)*** | ***8.441 (0.004)*** | ***2.348 (0.127)*** |  |
| Phonetic fluency ‘f’ | | F | -0.00 (0.92) | -0.22 (1.04) | -0.22 (0.86) | 1.196 (0.304) |
|  | | M | -0.00 (1.01) | -0.37 (1.10) | -0.74 (1.00) |  |
| ***Within-group sex effect, test stat (P value)*** | |  | ***0.000 (0.999)*** | ***0.719 (0.397)*** | ***4.466 (0.036)*** |  |
| SDMT | | F | 0.01 (0.88) | -0.38 (0.88) | -0.21 (0.80) | 4.040 (0.019)^a, b, c^ |
|  | | M | 0.01 (1.07) | -0.62 (0.85) | -1.10 (1.12) |  |
| ***Within-group sex effect, test stat (P value)*** | |  | ***0.000 (1.000)*** | ***1.906 (0.169)*** | ***14.596 (<0.001)*** |  |
| LNS | | F | 0.01 (0.89) | -0.03 (0.90) | -0.42 (0.85) | 0.522 (0.594) |
|  | | M | 0.01 (1.06) | -0.34 (1.07) | -0.62 (0.94) |  |
| ***Within-group sex effect, test stat (P value)*** | |  | ***0.000 (0.998)*** | ***2.911 (0.089)*** | ***0.756 (0.385)*** |  |
| JLO | | F | 0.00 (1.12) | -0.17 (1.07) | -0.28 (1.36) | 0.811 (0.446) |
|  | | M | 0.00 (0.82) | -0.12 (1.03) | -0.64 (1.27) |  |
| ***Within-group sex effect, test stat (P value)*** | |  | ***0.000 (1.000)*** | ***0.083 (0.773)*** | ***1.768 (0.185)*** |  |
| HLVT-R | |  |  |  |  |  |
|  | Immediate recall | F | -0.00 (0.90) | -0.27 (1.04) | -0.45 (0.89) | 0.226 (0.798) |
|  |  | M | -0.00 (1.00) | -0.17 (1.19) | -0.56 (1.07) |  |
|  | ***Within-group sex effect, test stat (P value)*** |  | ***0.000 (.999)*** | ***0.311 (0.577)*** | ***0.171 (0.679)*** |  |
|  | Recognition | F | -0.02 (0.85) | -0.13 (0.97) | -0.21 (0.84) | 0.070 (0.932) |
|  |  | M | -0.02 (1.08) | -0.09 (0.96) | -0.28 (0.97) |  |
|  | ***Within-group sex effect, test stat (P value)*** |  | ***0.000 (0.996)*** | ***0.047 (0.828)*** | ***0.094 (0.760)*** |  |
|  | Delayed recall | F | 0.02 (0.79) | -0.36 (1.03) | -0.50 (1.03) | 0.438 (0.646) |
|  |  | M | 0.02 (1.07) | -0.19 (1.13) | -.063 (1.10) |  |
|  | ***Within-group sex effect, test stat (P value)*** |  | ***0.250 (0.618)*** | ***0.745 (0.389)*** | ***0.250 (0.618)*** |  |

**Supplementary Table 4**

Neuropsychological tasks in z-scores adjusted by age and sex of HC, PD-non pRBD, and PD-pRBD females and males

Data are presented by groups as mean (SD). Neuropsychological variables are presented in z-scores adjusted by age and sex. Two-way analyses of variance (ANOVA), followed by Bonferroni post-hoc tests, were used for all variables.

^a^ Differences between HC males and PD-non pRBD males, ^b^ Differences between HC males and PD-pRBD males, ^c^ Differences between PD-non pRBD males and PD-pRBD males (P < 0.05).

Abbreviations: F= female; HC = healthy controls; HVLT-R = Hopkins Verbal Learning Test-Revised; JLO = Benton Judgment of Line Orientation; LNS = Letter-Number Sequencing; M = male; MoCA = Montreal Cognitive Assessment; PD-non pRBD = PD without probable RBD; PD-pRBD = PD with probable RBD; SDMT = Symbol Digit Modalities Test.

**Supplementary Table 5**

Between-group differences regarding the within-group sex effects in those z-scored variables adjusted by age and sex that showed group-by-sex interaction

|  | **Group-by sex**  **interaction**  **test stat (P value)** | **Contrast 1**  **HC vs. PD-non pRBD**  **(P value)** | **Contrast 2**  **HC vs. PD-pRBD**  **(P value)** | **Contrast 3**  **PD-non pRBD vs. PD-pRBD**  **(P value)** |
| --- | --- | --- | --- | --- |
| **Neuropsychological tasks** |  | | | |
| MoCA | 4.828 (0.009) | 0.501 | 0.003 | 0.022 |
| SDMT | 4.040 (0.019) | 0.411 | 0.007 | 0.025 |
| **Deep GM nuclei** |  | | | |
| Pallidum | 3.159 (0.044) | 0.796 | 0.058 | 0.016 |

Post-hoc contrasts were applied after two-way analysis of variance (ANOVA) of z-scored variables adjusted by age and sex.

Contrast 1 compare the sex effects between HC (HC males — HC females) and PD-non pRBD (PD-non pRBD males — PD-non pRBD females) groups.

Contrast 2 compare the sex effects between HC (HC males — HC females) and PD-pRBD (PD-pRBD males — pRBD females) groups.

Contrast 3 compare the sex effects between PD-non pRBD (PD-non pRBD males — PD-non pRBD females) and PD-pRBD (PD-pRBD males — PD-pRBD females) groups.

Abbreviations: GM = gray matter; HC = healthy controls; MoCA = Montreal Cognitive Assessment; PD-non pRBD = PD without probable RBD; PD-pRBD = PD with probable RBD; SDMT = Symbol Digit Modalities Test.

**Supplementary Table 6**

Magnetic resonance imaging derived measures in z-scores adjusted by age and sex of HC, PD-non pRBD, and PD-pRBD females and males

|  |  | **HC** | **PD-non pRBD** | **PD-pRBD** | **Group-by-sex**  **test stat (P value)** |
| --- | --- | --- | --- | --- | --- |
| **Global atrophy** |  |  |  |  |  |
| Cortical GM | F | 0.01 (0.85) | -0.32 (1.03) | 0.01 (1.09) | 1.363 (0.258) |
|  | M | 0.01 (1.08) | -0.59 (0.92) | -0.57 (1.14) |  |
| ***Within-group sex effect, test stat (P value)*** |  | ***0.000 (0.997)*** | ***2.037 (0.155)*** | ***5.457 (0.020)*** |  |
| Subcortical GM | F | 0.00 (0.97) | -0.18 (0.88) | 0.02 (1.12) | 1.771 (0.172) |
|  | M | 0.00 (0.93) | -0.16 (0.79) | -0.44 (0.84) |  |
| ***Within-group sex effect. test stat (P value)*** |  | ***0.000 (1.000)*** | ***0.012 (0.914)*** | ***4.587 (0.033)*** |  |
| Mean CTh, mm | F | 0.02 (0.85) | -0.12 (0.84) | -0.19 (0.73) | 0.011 (0.989) |
|  | M | 0.02 (1.08) | -0.16 (0.98) | -0.21 (1.06) |  |
| ***Within-group sex effect, test stat (P value)*** |  | ***0.000 (0.996)*** | ***0.058 (0.809)*** | ***0.007 (0.936)*** |  |
| **Deep GM nuclei** |  |  |  |  |  |
| Thalamus | F | 0.01 (0.78) | 0.15 (1.00) | 0.08 (1.00) | 0.751 (0.473) |
|  | M | 0.01 (1.00) | -0.09 (0.87) | -0.29 (0.78) |  |
| ***Within-group sex effect, test stat (P value)*** |  | ***0.000 (0.996)*** | ***2.179 (0.141)*** | ***2.900 (0.090)*** |  |
| Caudate | F | 0.00 (1.09) | -0.02 (1.05) | 0.30 (0.86) | 2.080 (0.127) |
|  | M | 0.00 (0.93) | -0.12 (0.98) | -0.32 (0.93) |  |
| ***Within-group sex effect, test stat (P value)*** |  | ***0.000 (1.000)*** | ***0.317 (0.574)*** | ***6.850 (0.009)*** |  |
| Putamen | F | -0.03 (1.05) | -0.28 (0.97) | -0.12 (1.15) | 1.085 (0.339) |
|  | M | -0.03 (0.90) | -0.17 (0.82) | -0.43 (0.94) |  |
| ***Within-group sex effect. test stat (P value)*** |  | ***0.000 (0.995)*** | ***0.375 (0.541)*** | ***1.877 (0.172)*** |  |
| Pallidum | F | -0,01 (0,95) | 0,05 (1,04) | 0,35 (1,01) | 3.159 (0.044) ^a^ |
|  | M | -0,02 (1,04) | 0,05 (1.04) | -0,31 (1,10) |  |
| ***Within-group sex effect. test stat (P value)*** |  | ***0.000 (0.997)*** | ***0.185 (0.667)*** | ***7.341 (0.007)*** |  |
| Hippocampus | F | 0.03 (0.97) | -0.36 (0.99) | -0.14 (1.05) | 0.759 (0.469) |
|  | M | 0.03 (0.88) | -0.17 (0.90) | -0.30 (0.91) |  |
| ***Within-group sex effect, test stat (P value)*** |  | ***0.000 (0.995)*** | ***1.184 (0.278)*** | ***0.489 (0.485)*** |  |
| Amygdala | F | -0.01 (0.95) | -0.54 (1.06) | -0.12 (1.64) | 2.253 (0.107) |
|  | M | -0.01 (1.03) | -0.34 (0.94) | -0.60 (1.00) |  |
| ***Within-group sex effect, test stat (P value)*** |  | ***0.000 (0.998)*** | ***1.076 (0.301)*** | ***3.492 (0.063)*** |  |
| Accumbens | F | 0.02 (1.19) | -0.13 (1.25) | 0.36 (1.29) | 1.507 (0.223) |
|  | M | 0.02 (0.82) | -0.045 (1.12) | -0.14 (1.18) |  |
| ***Within-group sex effect, test stat (P value)*** |  | ***0.000 (0.997)*** | ***0.156 (0.693)*** | ***3.291 (0.071)*** |  |
| Brainstem | F | -0.03 (0.84) | 0.22 (1.05) | 0.25 (0.91) | 2.113 (0.123) |
|  | M | -0.03 (1.11) | -0.17 (0.92) | -0.46 (1.05) |  |
| ***Within-group sex effect. test stat (P value)*** |  | ***0.000 (0.995)*** | ***4.752 (0.030)*** | ***8.518 (0.004)*** |  |

Data are presented by groups as mean (SD). Volumetric variables are presented in z-scores adjusted by age and sex. Two-way analyses of variance (ANOVA), followed by Bonferroni post-hoc tests, were used for all variables.

^a^ Differences between PD-non pRBD males and PD-pRBD males (P < 0.05).

Abbreviations: CTh = cortical thickness; F= female; GM = gray matter; HC = healthy controls; M = male; PD-non pRBD = PD without probable RBD; PD-pRBD = PD with probable RBD.

**Supplementary Table 7**

|  | |  | **PD-non pRBD** | **PD-pRBD** | **Group-by-sex**  **test stat (P value)** |
| --- | --- | --- | --- | --- | --- |
| MoCA | | F | -0.77 (1.81) | -0.11 (1.52) | 4.175 (0.042) |
|  | | M | -1.08 (1.93) | -1.56 (1.81) |  |
| Semantic fluency | |  |  |  |  |
|  | Animals | F | -0.23 (0.94) | -0.39 (0.75) | 1.837 (0.177) |
|  |  | M | -0.12 (0.97) | -0.66 (0.84) |  |
|  | Vegetables | F | 0.36 (1.19) | 0.09 (0.88) | 0.080 (0.778) |
|  |  | M | -0.58 (1.05) | -0.76 (1.06) |  |
|  | Fruits | F | 0.03 (0.95) | -0.11 (0.80) | 0.292 (0.590) |
|  |  | M | -0.51 (0.96) | -0.78 (0.95) |  |
| Phonetic fluency ‘f’ | | F | -0.34 (1.04) | -0.25 (0.84) | 2.158 (0.143) |
|  | | M | -0.31 (1.03) | -0.62 (0.95) |  |
| SDMT | | F | -0.43 (0.98) | -0.34 (0.86) | 3.061 (0.082) |
|  | | M | -0.58 (0.89) | -1.10 (1.11) |  |
| LNS | | F | -0.17 (0.92) | -0.61 (0.88) | 0.294 (0.588) |
|  | | M | -0.23 (1.09) | -0.55 (0.95) |  |
| JLO | | F | -0.52 (1.08) | -0.65 (1.39) | 1.062 (0.304) |
|  | | M | 0.14 (1.03) | -0.39 (1.27) |  |
| HLVT-R | |  |  |  |  |
|  | Immediate recall | F | 0.10 (1.03) | -0.07 (0.89) | 0.276 (0.600) |
|  |  | M | -0.43 (1.18) | -0.82 (1.07) |  |
|  | Recognition | F | -0.01 (0.90) | -0.01 (0.76) | 0.187 (0.666) |
|  |  | M | -0.17 (0.91) | -0.31 (0.98) |  |
|  | Delayed recall | F | -0.13 (0.94) | -0.18 (0.94) | 0.918 (0.339) |
|  |  | M | -0.43 (1.04) | -0.82 (1.09) |  |

Neuropsychological tasks scores of PD-non pRBD and PD-pRBD females and males

Data are presented in z-scores by groups as mean (SD). Two-way analyses of covariance (ANCOVA) with MDS-UPDRS part III as covariable were used for all variables.

Abbreviations: F= female; HC = healthy controls; HVLT-R = Hopkins Verbal Learning Test-Revised; JLO = Benton Judgment of Line Orientation; LNS = Letter-Number Sequencing; M = male; MoCA = Montreal Cognitive Assessment; PD-non pRBD = PD without probable RBD; PD-pRBD = PD with probable RBD; SDMT = Symbol Digit Modalities Test.

**Supplementary Table 8**

Magnetic resonance imaging derived measures of PD-non pRBD and PD-pRBD females and males

|  |  | **PD-non pRBD** | **PD-pRBD** | **Group-by-sex**  **test stat (P value)** |
| --- | --- | --- | --- | --- |
| **Global atrophy** |  |  |  |  |
| Cortical GM | F | 29.39 (2.23) | 30.03 (2.34) | 0.856 (0.356) |
|  | M | 28.15 (1.98) | 28.15 (2.39) |  |
| Subcortical GM | F | 3.63 (0.26) | 3.66 (0.32) | 2.429 (0.121) |
|  | M | 3.51 (0.24) | 3.41 (0.24) |  |
| Mean CTh, mm | F | 2.42 (0.10) | 2.41 (0.09) | 0.028 (0.866) |
|  | M | 2.39 (0.12) | 2.38 (0.12) |  |
| **Deep GM nuclei** |  |  |  |  |
| Thalamus | F | 0.465 (0.042) | 0.456 (0.044) | 0.209 (0.865) |
|  | M | 0.441 (0.040) | 0.429 (0.037) |  |
| Caudate | F | 0.221 (0.027) | 0.229 (0.022) | 2.875 (0.092) |
|  | M | 0.212 (0.025) | 0.207 (0.024) |  |
| Putamen | F | 0.294 (0.035) | 0.296 (0.039) | 1.473 (0.226) |
|  | M | 0.286 (0.029) | 0.275 (0.035) |  |
| Pallidum | F | 0.127 (0.014) | 0.131 (0.015) | 6.267 (0.013) ^a^ |
|  | M | 0.127 (0.013) | 0.120 (0.015) |  |
| Hippocampus | F | 0.267 (0.030) | 0.272 (0.031) | 1.122 (0.291) |
|  | M | 0.252 (0.028) | 0.247 (0.025) |  |
| Amygdala | F | 0.103 (0.015) | 0.108 (0.022) | 2.397 (0.046) |
|  | M | 0.105 (0.013) | 0.101 (0.013) |  |
| Accumbens | F | 0.031 (0.007) | 0.034 (0.007) | 2.180 (0.141) |
|  | M | 0.030 (0.006) | 0.030 (0.006) |  |
| Brainstem | F | 1.412 (0.128) | 1.412 (0.110) | 1.066 (0.303) |
|  | M | 1.375 (0.113) | 1.338 (0.128) |  |

Data are presented by groups as mean (SD). Volumetric variables are presented in ratios estimated by ((volume / eTIV) * 100). Two-way analyses of covariance (ANCOVA) with MDS-UPDRS part III as covariable were used for all variables.

^a^ Differences between PD-non pRBD males and PD-pRBD males (P < 0.05).

Abbreviations: CTh = cortical thickness; F= female; GM = gray matter; HC = healthy controls; M = male; PD-non pRBD = PD without probable RBD; PD-pRBD = PD with probable RBD.


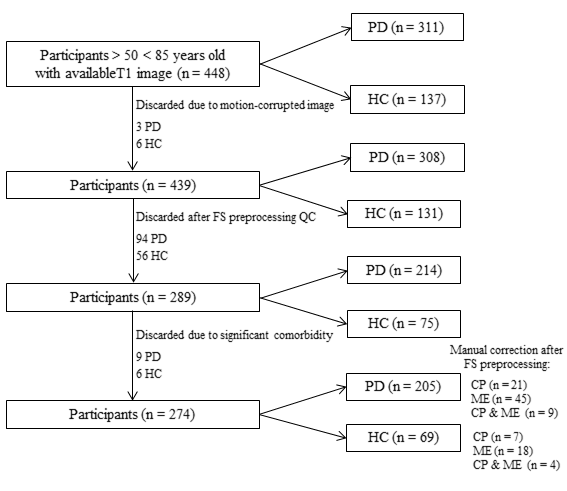
**Supplementary Figure 1.** Flow diagram of sample selection. Abbreviations: CP = control points; FS = FreeSurfer; HC = healthy controls; ME = manual erase; PD = Parkinson’s disease; QC = quality control.
